# Innovative 3D printing technologies and advanced materials revolutionizing orthopedic surgery: current applications and future directions

**DOI:** 10.3389/fbioe.2025.1542179

**Published:** 2025-02-11

**Authors:** Bo Cong, Haiguang Zhang

**Affiliations:** ^1^ Department of Orthopedics, Yantaishan Hospital Affiliated to Binzhou Medical University, Yantai, Shandong, China; ^2^ Yantai Key Laboratory for Repair and Reconstruction of Bone and Joint, Yantai, Shandong, China

**Keywords:** 3D printing, technology, material, challenge, FGM

## Abstract

Three-dimensional (3D) printing has rapidly become a transformative force in orthopedic surgery, enabling the creation of highly customized and precise medical implants and surgical tools. This review aims to provide a more systematic and comprehensive perspective on emerging 3D printing technologies—ranging from extrusion-based methods and bioink printing to powder bed fusion—and the broadening array of materials, including bioactive agents and cell-laden inks. We highlight how these technologies and materials are employed to fabricate patient-specific implants, surgical guides, prosthetics, and advanced tissue engineering scaffolds, significantly enhancing surgical outcomes and patient recovery. Despite notable progress, the field faces challenges such as optimizing mechanical properties, ensuring structural integrity, addressing regulatory complexities across different regions, and considering environmental impacts and cost barriers, especially in low-resource settings. Looking ahead, innovations in smart materials and functionally graded materials (FGMs), along with advancements in bioprinting, hold promise for overcoming these obstacles and expanding the capabilities of 3D printing in orthopedics. This review underscores the pivotal role of interdisciplinary collaboration and ongoing research in harnessing the full potential of additive manufacturing, ultimately paving the way for more effective, personalized, and durable orthopedic solutions that improve patient quality of life.

## 1 Introduction

Orthopedic surgery has experienced significant advancements in recent years, propelled by breakthroughs in surgical techniques and materials science. The fundamental goal of orthopedic interventions is to restore mobility, enhance functionality, and alleviate pain associated with musculoskeletal disorders (Liang W. et al., 2024; Smeeing et al., 2017). Traditional orthopedic approaches typically employ standardized implants designed to fit a broad range of anatomical structures. However, this one-size-fits-all methodology often falls short in addressing the unique anatomical variations of individual patients, leading to complications such as implant misalignment, premature failure, and prolonged recovery periods (Marin and Lanzutti, 2023).

A diverse array of materials is utilized in orthopedic implants, each chosen for its specific properties that facilitate bone healing and integration (Castrisos et al., 2022; Valente et al., 2022). Metals, particularly titanium and stainless steel, are favored for their exceptional strength, durability, and biocompatibility, making them ideal for load-bearing applications such as joint replacements and spinal implants (Ozdemir et al., 2022). Nevertheless, the long-term use of metallic implants can result in adverse effects, including inflammation, swelling, and discomfort, primarily due to ion leaching and wear-induced debris (Rajaeirad et al., 2024). These drawbacks have catalyzed a shift towards the incorporation of polymers in orthopedic applications. Polymers, such as Polylactic Acid (PLA) and Polyether Ether Ketone (PEEK), are increasingly replacing traditional metallic components like bone fixation plates and screws (Al-Shalawi et al., 2023; S et al., 2024). Their use not only mitigates metal-on-metal contact in joint replacements—thereby reducing the risk of inflammatory responses—but also offers benefits in terms of lighter weight and enhanced biocompatibility. Additionally, ceramics are employed in scenarios requiring materials with specific biocompatibility and wear resistance characteristics, further broadening the spectrum of available options to meet diverse clinical demands (Lodge et al., 2023; Skriabin et al., 2022; Vaiani et al., 2023).

The advent of three-dimensional (3D) bioprinting, also known as additive manufacturing, has introduced a transformative paradigm in the design and fabrication of orthopedic implants. Unlike conventional manufacturing techniques, 3D printing enables patient-specific design, offers enhanced geometric complexity, and supports the fabrication of structures that closely mimic native bone architectures. Furthermore, bioink-based extrusion and cell-laden printing approaches allow the direct incorporation of living cells and bioactive agents within scaffolds, fostering improved tissue integration (Murphy and Atala, 2014; Wan et al., 2020). This level of personalization is particularly advantageous in overcoming the limitations of standardized implants, ensuring optimal fit and integration with the patient’s bone tissue. Moreover, 3D printing facilitates the creation of complex geometries and porous structures that promote biological integration and bone ingrowth—features that are challenging to achieve with traditional manufacturing methods. These advancements not only enhance the mechanical compatibility of implants but also support the biological processes essential for long-term success and stability (Gharibshahian et al., 2023; Goldsmith et al., 2020).

Beyond personalization, 3D printing surpasses many conventional manufacturing techniques in complexity handling, design flexibility, and material efficiency, potentially reducing healthcare costs by minimizing the need for revision surgeries and accelerating patient recovery times. The ability to rapidly produce patient-specific implants and surgical tools streamlines the surgical workflow, reduces operative time, and enhances overall surgical precision (Yaneva et al., 2023; Zahid et al., 2024). Comparative analyses show that while conventional machining or forging can produce standardized implants at scale, they often lack the precision and patient specificity that 3D printing offers.

Despite these advantages, gaps remain. The long-term durability of certain 3D-printed materials under dynamic physiological loads, anisotropy in mechanical properties, and limited regulatory frameworks across regions complicate clinical translation. Furthermore, environmentally sustainable materials and processes, as well as strategies to lower costs in resource-limited settings, are increasingly relevant considerations. As 3D printing technology continues to evolve, its applications in orthopedic surgery are expanding, paving the way for innovative treatment modalities that were previously unattainable. The integration of advanced materials, computational modeling, AI-driven design optimization, and sophisticated manufacturing techniques holds promise for the development of next-generation orthopedic solutions that deliver superior clinical outcomes and improved quality of life for patients.

**FIGURE 1:**
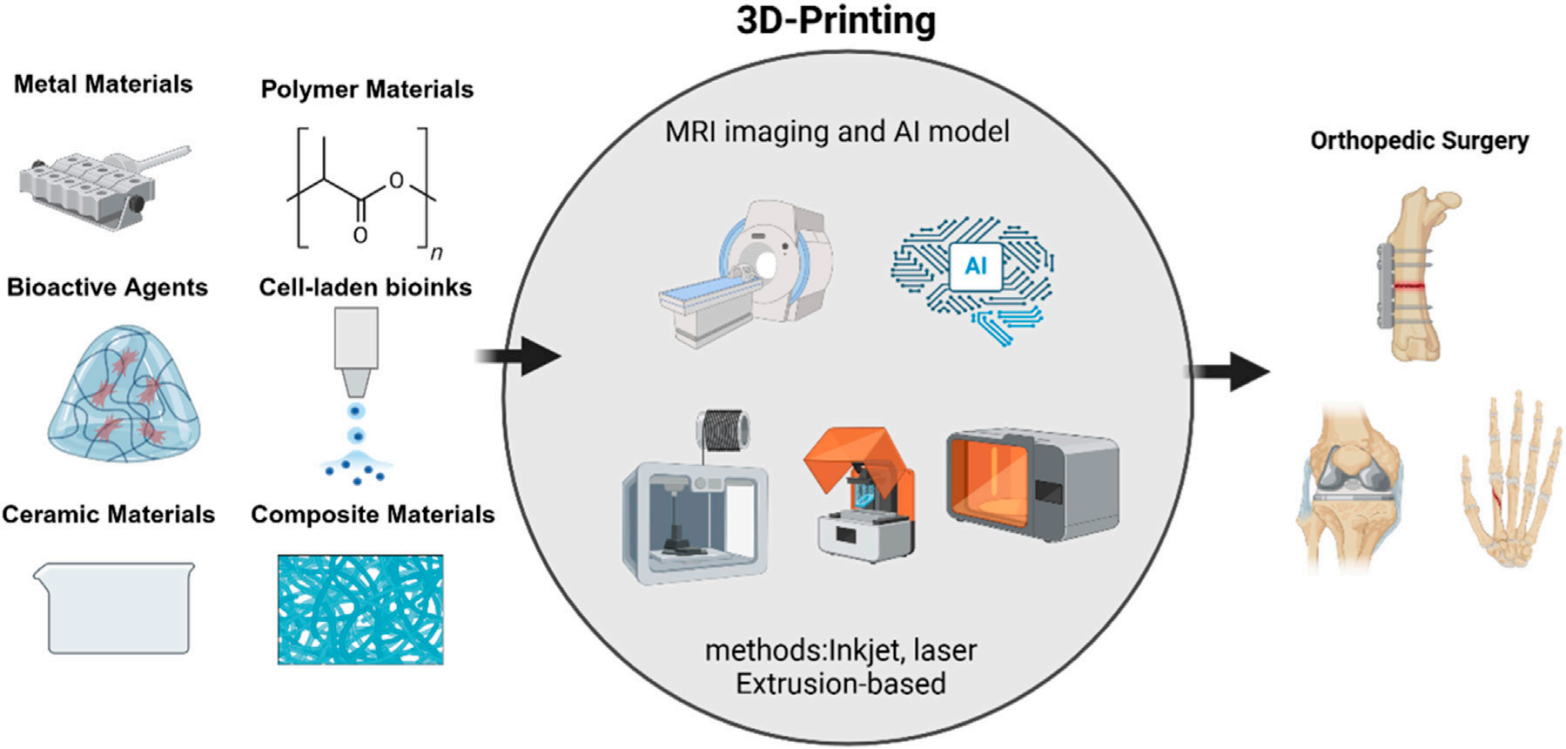
Comprehensive overview of 3D printing in orthopedic surgery.

This review provides a systematic overview of key 3D printing technologies, including material extrusion (e.g., FDM), vat photopolymerization (SLA), powder bed fusion techniques (SLS and DMLS), and emerging bioink-based methods. We also detail the expanded classification of biomaterials and bioactive agents, discuss current clinical trials and barriers to translation, and highlight ongoing regulatory challenges. By connecting these advances to specific clinical problems—such as precision bone regeneration and complex joint reconstructions—we underline the direct benefits to orthopedic surgery (Figure 1).

## 2 3D printing technologies overview in orthopedics

Several 3D printing technologies are commonly employed in orthopedic applications, each with distinct advantages, limitations, and suitable use cases. The primary technologies include Stereolithography (SLA), Selective Laser Sintering (SLS), Fused Deposition Modeling (FDM), and Direct Metal Laser Sintering (DMLS). There is growing interest in material extrusion processes (including direct ink writing) and bioink printing techniques. Moreover, powder bed fusion technologies have broadened beyond polymer-based SLS to include various metal and ceramic printing approaches. This section provides a comprehensive overview of these methods and highlights their unique mechanisms and contributions to orthopedic solutions (Table 1).

**TABLE 1:** Comparative summary of 3D printing technologies in orthopedics.

Technology	Mechanism	Common materials	Orthopedic applications	Advantages	Limitations
SLA	Laser cures liquid photopolymer layer by layer	Photopolymer resins	High-precision models, surgical guides	High resolution, smooth surfaces	Limited mechanical strength, post-curing required, not load-bearing
SLS	Laser sinters polymer powder (powder bed fusion)	Nylon, TPU, bioresorbable polymers	Prosthetic components, spinal cages, instruments	No support structures, versatile materials	Rough surface, lower resolution than SLA
FDM	Thermoplastic filament extrusion layer by layer	PLA, ABS, PEEK	Anatomical models, low-load implants, prototypes	Cost-effective, easy to operate	Visible layer lines, mechanical properties depend on print settings
DMLS	Laser/e-beam melts/fuses metal powder (powder bed fusion)	Titanium alloys, stainless steel, Co-Cr	Custom metal implants (joints, spine), high-load	High strength, complex geometry	Expensive, extensive post-processing, specialized equipment
Material Extrusion	Viscous pastes/slurries deposited at ambient/low T	Ceramic or polymer-based pastes	Porous bone scaffolds, composite implants	Can print multi-phase materials, tailored porosity	Typically lower resolution, may need post-sintering or curing
Bioink Printing	Deposits cell-laden or bioactive inks (extrusion/inkjet)	Hydrogels (e.g., alginate, GelMA), cells, growth factors	Tissue engineering (bone/cartilage), regenerative scaffolds	Enables living cells, personalized constructs	Low mechanical strength, maintaining cell viability is challenging

### 2.1 Stereolithography

SLA stands as one of the pioneering 3D printing technologies, employing a laser to selectively cure liquid photopolymer resins layer by layer (Stoia et al., 2019). In the field of orthopedics, SLA is primarily utilized for fabricating high-precision anatomical models, surgical guides, and bespoke implants (Matter-Parrat and Liverneaux, 2019; Husna et al., 2024). The exceptional resolution and smooth surface finish achievable with SLA make it ideal for applications that demand intricate details and exact dimensions. Compared to other 3D printing techniques, SLA provides superior accuracy, facilitating the creation of highly detailed and precise models. The laser curing process minimizes surface roughness, thereby reducing the necessity for extensive post-processing (Chan et al., 2010). However, SLA predominantly relies on photopolymers, which may not possess the mechanical strength required for load-bearing orthopedic implants. Additionally, the printed components often require further curing and the removal of support structures, which can increase both production time and complexity (Stoia et al., 2019; Arcaute et al., 2006; Chen et al., 2010; Ganz, 2005). Ultimately, SLA is best suited for developing detailed anatomical replicas for pre-surgical planning, patient-specific surgical guides, and prototypes where high precision is paramount.

### 2.2 Selective laser sintering

SLS is an advanced additive manufacturing technique that utilizes a laser to sinter powdered materials, predominantly polymers, layer by layer to construct solid and intricate structures (Chang et al., 2017). In the realm of orthopedics, SLS is instrumental in producing robust and complex implants, prosthetics, and surgical instruments. Its capacity to handle a diverse array of polymers, including biocompatible and bioresorbable materials, renders SLS highly adaptable for various orthopedic applications.

One of the primary advantages of SLS is its material versatility. The technology can process a wide range of polymers such as nylon and other biocompatible substances, facilitating the creation of diverse orthopedic devices tailored to specific clinical needs (Du et al., 2017; Rahmani et al., 2023; Yu et al., 2022). Additionally, SLS eliminates the necessity for support structures during the printing process. The unsintered powder surrounding the printed object acts as a natural support, allowing for the fabrication of complex geometries without the need for additional materials, thereby simplifying the manufacturing process.

However, SLS does present certain limitations. A common drawback is the surface roughness of the printed parts, which often requires post-processing to achieve the desired finish for specific medical applications. Furthermore, while SLS is versatile, it generally offers lower resolution compared to SLA. This reduced resolution can limit its effectiveness in applications that demand fine details and high precision (Msallem et al., 2024).

Despite these challenges, SLS remains ideal for producing functional orthopedic implants such as spinal cages and joint replacements. Its ability to create durable prosthetic devices with complex geometries and significant mechanical strength makes it a valuable tool in orthopedic manufacturing. The combination of material versatility and the capability to fabricate intricate designs ensures that SLS continues to play a crucial role in advancing orthopedic solutions.

### 2.3 Fused deposition modeling

Material extrusion encompasses a family of techniques where a thermoplastic filament, paste, or gel is selectively deposited through a nozzle to form layers. FDM constructs parts by extruding thermoplastic filaments through a heated nozzle, layer by layer (Acierno and Patti, 2023). In orthopedics, FDM is employed to create patient-specific anatomical models, surgical planning tools, and prototypes of implants and prosthetic devices. Its affordability and accessibility make FDM an attractive option for early design stages and educational purposes (Winarso et al., 2022).

A significant advantage of FDM is its cost-effectiveness, as both printers and materials are generally less expensive compared to other 3D printing technologies, enhancing its accessibility for various applications. Additionally, FDM supports a broad range of thermoplastics, including Polylactic Acid (PLA), Acrylonitrile Butadiene Styrene (ABS), and Polyether Ether Ketone (PEEK), providing flexibility in material selection to meet different mechanical and biocompatibility requirements in orthopedic applications (Jaber et al., 2021; Kumar et al., 2020; Eichholz et al., 2024).

However, FDM has its limitations. It typically produces parts with visible layer lines and lower dimensional accuracy, resulting in a rough surface finish that often requires post-processing for high-fidelity applications. Moreover, the mechanical properties of FDM-printed parts are highly dependent on printing parameters such as layer height, print speed, and infill density, which can affect the strength and durability of the final product, potentially limiting its suitability for load-bearing implants (Wickramasinghe et al., 2020).

Despite these challenges, FDM remains well-suited for specific scenarios within orthopedics. It is particularly effective for creating educational models, surgical planning tools, and prototypes where cost-efficiency and rapid production are prioritized over high precision and surface quality. The ability to quickly generate customized models supports improved surgical planning and educational outcomes, making FDM a valuable tool in both clinical and academic settings. Moreover, newer variants of extrusion-based printing, such as direct ink writing (DIW) or robocasting, allow the extrusion of more viscous materials (e.g., ceramic slurries or polymer pastes) at room or slightly elevated temperatures (Shahzad and Lazoglu, 2021). These emerging methods open the door to multi-material scaffolds and custom porous architectures that can be fine-tuned for load-bearing or regenerative orthopedic applications.

### 2.4 Direct metal laser sintering

DMLS is an advanced 3D printing technology that fabricates dense and robust metal components by sintering metal powders with a laser, layer by layer. In orthopedics, DMLS is essential for producing intricate metal implants, including titanium and stainlesss teel joint replacements, spinal implants, and custom prosthetic devices (Grecchi et al., 2020). Its ability to achieve superior mechanical properties and high customization makes DMLS ideal for critical orthopedic applications.

A primary advantage of DMLS is the exceptional mechanical strength of its metal components, which are comparable to traditionally manufactured implants, making them suitable for load-bearing uses. Additionally, DMLS enables the creation of complex, patient-specific implant designs that are challenging to achieve with conventional manufacturing methods, allowing for implants tailored to individual anatomical and functional needs (Jardini et al., 2014; Roos et al., 2020). Nevertheless, DMLS has some drawbacks. The technology and metal powders required are relatively expensive, increasing the cost of producing orthopedic implants. Furthermore, DMLS-fabricated metal parts often need extensive post-processing, such as heat treatment and surface finishing, to achieve the desired mechanical properties and surface quality, which adds to production time and complexity (Davoodi et al., 2023). In addition to SLS (polymers) and DMLS (metals), other variants include Selective Laser Melting (SLM) and Electron Beam Melting (EBM), both of which can be used with various metals or alloys. EBM, for example, employs an electron beam under vacuum, minimizing oxidation and potentially reducing material stresses (Shi et al., 2023). These extended powder bed fusion methods enable tighter control over microstructure, offering improved mechanical properties, enhanced fatigue resistance, and the ability to create highly porous, patient-specific scaffolds. As a result, SLM and EBM are increasingly considered for high-performance orthopedic implants, especially in demanding load-bearing sites where design complexity and structural integrity are paramount.

Despite these challenges, DMLS remains the preferred method for manufacturing custom metal implants in orthopedics. Its capability to produce implants with high mechanical strength, biocompatibility, and complex geometries supports applications like patient-specific joint replacements and spinal implants. The precision and customization provided by DMLS enhance clinical outcomes and patient satisfaction, underscoring its essential role in advancing orthopedic solutions.

### 2.5 Bioink printing

Bioink printing refers to the deposition of cell-laden or bioactive inks that incorporate living cells, growth factors, and biocompatible polymers. This approach enables the fabrication of scaffolds with built-in biological functionality, which is especially valuable in orthopedics for engineering bone and cartilage tissue constructs (Chen et al., 2023). Techniques such as extrusion-based bioprinting, droplet-based bioprinting, and laser-assisted bioprinting are used to precisely place cells and supportive hydrogel matrices, creating constructs that mimic the extracellular matrix and foster cellular integration (Ma et al., 2024). While many bioink formulations focus on hydrogels like alginate, collagen, or GelMA, recent advancements include composite bioinks containing ceramic particles (e.g., hydroxyapatite) for improved osteoconductivity. Despite ongoing challenges with maintaining cell viability during printing and achieving sufficient mechanical strength for load-bearing applications, bioink-based methods hold enormous promise for patient-specific regenerative therapies in complex orthopedic defects (Badhe et al., 2023).

## 3 Materials classification and characteristics

In the realm of orthopedic 3D printing, the selection of appropriate materials is paramount to ensure the functionality, biocompatibility, and longevity of medical implants and devices. Materials used in this field can be broadly categorized into metals, polymers, ceramics, and composites, each offering unique properties that cater to specific clinical requirements. Besides, the role of bioactive agents and cell-laden inks has also become increasingly important for promoting enhanced bone regeneration and tissue integration. Understanding the distinct characteristics and applications of these material classes is essential for advancing orthopedic solutions through additive manufacturing (Table 2).

**TABLE 2:** Classification and characteristics of 3D printing materials in orthopedics.

Material category	Examples	Key properties	Orthopedic applications	Advantages	Limitations
Metals	Titanium alloys, stainless steel, Co-Cr	High strength, durable, biocompatible	Load-bearing implants (joints, spine)	Superior mechanical performance, long-term use	Expensive powders, stress shielding, post-processing required
Polymers	PLA, ABS, PEEK, PCL, SMPs (PU)	Lightweight, tunable mechanical properties, some biodegradable	Models, guides, low- to medium-load implants	Cost-effective, easy to fabricate	May lack strength for heavy load, thermal/chemical stability issues
Ceramics	Hydroxyapatite, alumina, zirconia	Excellent biocompatibility, wear-resistant, brittle	Bone grafts, coatings, joint surfaces	Promotes bone ingrowth, low wear	Brittle under impact, high-temp processing, polishing often needed
Composites	Metal-polymer, ceramic-polymer, fiber-reinforced	Combined strengths of constituents (flexibility + strength)	Complex implants needing multiple properties	Tailorable properties, can reduce stress shielding	More complex fabrication, bonding challenges, higher costs
Bioactive and cell-laden	Growth factors (BMPs), antimicrobial agents, hydrogel-cell inks	Enhance osteogenesis, infection control, tissue integration	Regenerative scaffolds, living implants	Accelerates healing, potential for custom biology	Low mechanical strength, regulatory hurdles, cell viability concerns

### 3.1 Metal materials

Metallic materials are extensively utilized in orthopedic 3D printing due to their superior mechanical strength, durability, and biocompatibility. Commonly used metals include titanium alloys, stainless steel, and cobalt-chromium alloys (Bandyopadhyay et al., 2023). Titanium alloys, such as Ti-6Al-4V, are favored for their excellent biocompatibility and favorable strength-to-weight ratio, making them ideal for load-bearing implants like joint replacements and spinal devices (Veiga et al., 2012; Osman and Swain, 2015). Stainless steel offers robust mechanical properties at a lower cost, suitable for temporary implants and surgical instruments. Cobalt-chromium alloys provide exceptional wear resistance and are often employed in applications requiring high durability, such as dental implants and long-term joint prostheses (Niinomi, 2008; Sarswat et al., 2020; Hollander et al., 2006). The ability of metal 3D printing techniques, particularly DMLS, to fabricate complex geometries and patient-specific designs enhances the customization and performance of orthopedic implants. Recent surface treatments and coatings have also emerged to address concerns such as ion leaching, wear debris, and suboptimal osseointegration. For instance, diamond-like carbon (DLC) coatings can reduce friction and mitigate metal ion release in load-bearing joints, (Riosalido et al., 2025; Shah et al., 2024; Roy et al., 2024). Moreover, techniques like plasma electrolytic oxidation (PEO) and anodization can create micro- and nanoporous surfaces on titanium alloys, improving cell adhesion and helping to regulate ion release (Fattah-alhosseini et al., 2024). Such innovations highlight the ongoing efforts to refine metallic implants for greater durability, biocompatibility, and long-term clinical performance, complementing the design freedom afforded by 3D printing.

Despite their advantages, metal materials present certain challenges. The high cost of metal powders and the complexity of processing require significant investment in equipment and expertise. Additionally, metal implants can be prone to stress shielding, where the stiffness of the implant may lead to bone resorption over time. Post-processing steps, including heat treatment and surface finishing, are often necessary to achieve the desired mechanical properties and surface characteristics, further increasing production time and costs (Balla et al., 2010). Nevertheless, the unparalleled mechanical performance and biocompatibility of metals ensure their continued prominence in critical orthopedic applications.

### 3.2 Polymer materials

Polymeric materials offer versatility and adaptability in orthopedic 3D printing, enabling the creation of both rigid and flexible components tailored to specific medical needs. Common polymers used include Polylactic Acid (PLA), Acrylonitrile Butadiene Styrene (ABS), and Polyether Ether Ketone (PEEK). PLA and ABS are frequently employed for producing anatomical models, surgical guides, and prototypes due to their ease of processing and cost-effectiveness (Nahan et al., 2020). PEEK, a high-performance polymer, is increasingly utilized for load-bearing implants such as spinal cages and cranial plates due to its excellent mechanical properties, chemical resistance, and biocompatibility (Giubilini et al., 2021). Additionally, bioresorbable polymers like Polycaprolactone (PCL) are used in tissue engineering applications, where temporary scaffolds support bone regeneration and gradually degrade as new tissue forms (Liang HY. et al., 2024; Siddiqui et al., 2018; Sowmya et al., 2021).

Recent developments in polymer science have significantly broadened the range of materials available for orthopedic applications. For instance, shape memory polymers (SMPs) such as polyurethane (PU) have emerged as promising candidates for implants that can adapt to dynamic physiological environments. These materials can be compressed into a temporary shape for minimally invasive insertion and then recover their original geometry when exposed to a specific stimulus, such as body temperature. A novel near-infrared-responsive shape memory scaffold comprising polyurethane and magnesium (fabricated via low-temperature rapid prototyping 3D printing) demonstrates excellent mechanical properties, stable photothermal effects, and the ability to quickly recover its original shape under near-infrared irradiation (Zhang et al., 2022). Such reversible transformations are particularly advantageous for complex procedures where an implant needs to conform precisely to patient-specific anatomical contours while also retaining robust mechanical performance post-deployment. By incorporating urethane-based PEGylated poly (glycerol sebacate) (PEGSU) into ceramic bioinks, the scaffold can be rapidly prototyped at low temperature, providing superior mechanical strength, hyperelasticity, and effective support for cell proliferation and osteogenic differentiation (Ma et al., 2021). In parallel, superelastomers like poly (glycerol sebacate) (PGS) are garnering attention due to their high elasticity and rapid recovery, which allow these polymers to endure repetitive loading without permanent deformation. Their rubber-like properties can be tuned to match the elasticity of soft tissues, thus minimizing interface mismatches in applications ranging from cartilage repair to flexible joint prostheses. A similarly active research area involves hydrogel-based systems, which are highly hydrated networks designed to emulate certain characteristics of native extracellular matrices. Examples include collagen or gelatin methacryloyl (GelMA) hydrogels, which are often combined with bioactive agents or living cells to enhance osteogenesis or chondrogenesis (Kurian et al., 2022). Their high water-content promotes cellular infiltration and nutrient diffusion, making them well suited for regenerative approaches in bone or cartilage repair. However, to address the mechanical limitations inherent in pure hydrogels, researchers are increasingly exploring composite strategies—for example, reinforcing hydrogel networks with ceramic particles or integrating short fiber fillers to achieve a balance of flexibility, biological functionality, and mechanical robustness. In doing so, hydrogels can provide an ideal niche for cell growth while meeting the structural demands of the orthopedic environment.

Moreover, the burgeoning field of multi-material 3D printing is opening new avenues for fabricating polymeric scaffolds that more closely mimic the hierarchical complexity of bone. By layering different polymers—such as a shape-memory polymer core with a hydrogel shell—researchers can create constructs with graduated mechanical and biological properties, improving tissue integration and potentially reducing stress shielding. This approach is particularly attractive for large defect sites where an implant must combine osteoconductive regions with supportive load-bearing areas. Ongoing advances also focus on incorporating nanostructures (e.g., carbon nanotubes, bioactive glass) to augment mechanical resilience and confer additional functionalities like antimicrobial or angiogenic properties (Chen and Li, 2022; Shar et al., 2023).

Taken together, these evolving strategies in polymer materials underscore the move toward implants and scaffolds that are not only biocompatible but can dynamically adapt to changing physiological conditions. Although regulatory considerations and scaling up production to clinical volumes remain challenges, the versatility and tunability of advanced polymer systems show considerable promise for elevating patient outcomes and facilitating customized therapeutic approaches in orthopedic medicine. While polymers provide significant advantages in terms of customization and ease of use, they also exhibit limitations. Many polymers lack the mechanical strength required for long-term load-bearing applications, necessitating the use of reinforced or composite materials for enhanced performance. The thermal and chemical stability of certain polymers can also be a concern, potentially affecting the integrity and longevity of the printed implants. Moreover, achieving precise mechanical properties often requires careful control of printing parameters and material formulations. Despite these challenges, the ongoing development of advanced polymers and composite formulations continues to expand the applicability of polymeric materials in orthopedic 3D printing.

### 3.3 Ceramic materials

Ceramic materials are renowned for their excellent biocompatibility, bioactivity, and wear resistance, making them ideal for specific orthopedic applications. Common ceramics used in 3D printing include hydroxyapatite (HA), alumina (Al₂O₃), and zirconia (ZrO₂) (Jayaswal et al., 2010; Piconi, 2017). Hydroxyapatite, a naturally occurring mineral form of calcium apatite, is extensively used for bone grafts and coatings on metal implants due to its ability to promote bone in-growth and osseointegration. Alumina and zirconia ceramics offer exceptional hardness and wear resistance, making them suitable for joint prostheses and bearing surfaces where low friction and high durability are critical (Palmero et al., 2014). The ability to fabricate porous ceramic structures through additive manufacturing facilitates bone tissue ingrowth and enhances the integration of implants with the surrounding bone. Despite their favorable properties, ceramic materials present certain drawbacks in orthopedic applications. Ceramics are inherently brittle, which can lead to fracture under impact or high-stress conditions, limiting their use in load-bearing applications without adequate support. Additionally, the processing of ceramics through 3D printing can be challenging due to their high melting temperatures and the need for precise control over sintering processes (Li et al., 2023; Zhao et al., 2023). Post-processing steps, such as grinding and polishing, are often required to achieve the desired surface finish and mechanical properties. Nevertheless, the unique advantages of ceramic materials in promoting bone regeneration and providing wear-resistant surfaces ensure their continued use in specialized orthopedic applications.

### 3.4 Composite materials

Composite materials, which combine two or more different material types, offer the ability to tailor properties to meet specific orthopedic requirements. By integrating metals, polymers, or ceramics with each other or with reinforcing agents such as carbon fibers or bioactive ceramics, composites can achieve a balance of mechanical strength, flexibility, and biocompatibility (Scholz et al., 2011). For instance, metal-polymer composites can provide the structural support of metals while incorporating the flexibility and bioactivity of polymers, making them suitable for applications like spinal implants and joint prostheses. Ceramic-polymer composites enhance bioactivity and wear resistance, ideal for bone scaffolds and coatings on implants to promote osseointegration and reduce wear-related complications (Ornaghi et al., 2023).

The primary advantage of composite materials lies in their ability to synergize the beneficial properties of their constituent materials, resulting in enhanced overall performance. However, the fabrication of composite materials through 3D printing presents technical challenges, including ensuring uniform material distribution and achieving strong interfacial bonding between different phases. Additionally, the complexity of processing multi-material composites can increase production time and cost. Despite these challenges, the versatility and superior performance of composite materials make them a promising avenue for advancing orthopedic 3D printing, offering customized solutions that address the diverse and evolving needs of patients.

### 3.5 Bioactive agents and cell-laden inks

Beyond traditional bulk materials, the orthopedic community has increasingly turned to bioactive agents (e.g., growth factors, antimicrobial peptides) and cell-laden inks (e.g., hydrogels containing osteoprogenitor or stem cells) as strategies to enhance tissue regeneration and implant integration. Bioactive molecules such as bone morphogenetic proteins (BMPs) have been incorporated into porous polymeric scaffolds to stimulate osteogenic differentiation at the defect site, while antimicrobial peptides (e.g., LL-37) embedded in hydrogel matrices can reduce the risk of postoperative infection by providing localized, sustained release (Tang et al., 2024; Zhu et al., 2022). By allowing these agents to be positioned precisely where they are needed, 3D printing can address challenges that conventional implants or systemic therapies often fail to resolve.

Cell-laden inks add another dimension to this paradigm, as they introduce living cells directly into the scaffold during the printing process. For example, printing a gelatin methacryloyl (GelMA) ink seeded with stem cells can create hybrid constructs that foster early osteogenesis or chondrogenesis *in situ* (Lv et al., 2023). Similarly, some researchers have combined extrusion-based bioprinting with stiffer polymers like polycaprolactone (PCL), creating composite implants that simultaneously offer mechanical support and a microenvironment conducive to cell survival and differentiation (Gharibshahian et al., 2023). Such multi-material printing approaches can even yield functionally graded implants, where certain regions prioritize cellular activity while others contribute to load-bearing capacity, a feature particularly relevant for large or irregular bone defects.

Despite these promising developments, cell-laden and bioactive scaffolds do face significant hurdles. One principal concern is mechanical robustness, as hydrogels or cell-laden constructs typically lack the strength required for high-load applications without additional reinforcement from inorganic particles or polymeric frameworks. Moreover, maintaining viable cells throughout the printing process demands optimized bioink rheology, careful temperature control, and gentle extrusion parameters, all of which can complicate production workflows. Coupled with the need for sterility and precise compositional consistency, these requirements underscore the complexities inherent to biofabrication.

### 3.6 Environmental considerations in orthopedic 3D printing

With growing attention to sustainability, the environmental impact of 3D-printed orthopedic devices is becoming an increasingly important topic. Metals (e.g., titanium, cobalt-chromium) often require energy-intensive powder production and post-processing steps, although the recyclability of metal powders can mitigate waste. In contrast, polymers like PLA or PCL offer biodegradable or bioresorbable options that reduce long-term material accumulation in the body but may still involve petrochemical feedstocks or limited recycling infrastructure (Zhu et al., 2025). Ceramics, prized for their biocompatibility, generally demand high-temperature sintering, leading to significant energy consumption during fabrication (Wang et al., 2023). Composite materials often involve multiple components that can be challenging to separate at end-of-life, complicating recyclability. Nonetheless, emerging manufacturing optimizations—such as more efficient powder reuse strategies, lower-temperature printing protocols, and the development of greener bio-based polymers—are gradually reducing the carbon footprint of additive manufacturing. Future research that integrates life-cycle assessments and closed-loop material processes will be crucial for achieving more eco-friendly, large-scale deployment of 3D printing in orthopedics.

## 4 Applications of 3D printing materials in orthopedics

The adoption of 3D printing technology in orthopedics has significantly transformed the landscape of medical device manufacturing and surgical procedures. By enabling the creation of highly customized and precise medical solutions, 3D printing enhances patient outcomes and surgical efficiency. This section explores the primary applications of 3D-printed materials in orthopedics, including the development of tailored implants, surgical guides, prosthetics, and tissue engineering scaffolds.

### 4.1 Customized implants

One of the most impactful applications of 3D printing in orthopedics is the fabrication of patient-specific implants. Traditional manufacturing methods often rely on standardized implant sizes, which may not perfectly conform to an individual’s unique anatomical structure, potentially compromising fit and functionality. Additive manufacturing allows for the production of implants that are precisely tailored to match the patient’s bone geometry, improving fit and promoting better integration with the surrounding bone tissue. For example, 3D-printed titanium implants have been successfully utilized in spinal surgeries, where complex geometries are necessary to support intricate spinal structures (Fan et al., 2015; Lewandrowski et al., 2024). Additionally, the incorporation of porous architectures within these implants enhances osseointegration, reducing the likelihood of implant loosening and improving long-term stability.

### 4.2 Surgical guides and models

3D printing also plays a crucial role in surgical planning and execution through the creation of accurate anatomical models and surgical guides. Preoperative planning using 3D-printed models allows surgeons to visualize and rehearse complex procedures, leading to increased surgical precision and reduced operative times. Surgical guides, which are custom-designed tools created based on patient-specific anatomical data, assist in accurately positioning implants and making precise bone cuts (Le Stum et al., 2023). Studies have demonstrated that the use of 3D-printed surgical guides in knee and hip replacement surgeries results in better alignment of implants and improved postoperative outcomes (Kumar et al., 2021). Furthermore, these guides facilitate minimally invasive surgical techniques, which can enhance patient recovery and reduce surgical trauma.

### 4.3 Prosthetics

The customization capabilities of 3D printing are particularly beneficial in the development of prosthetic limbs. Traditional prosthetics can be expensive and time-consuming to produce, often lacking the necessary personalization for individual users. 3D printing enables the rapid production of lightweight, durable, and highly customized prosthetic devices that conform to the unique anatomical and functional requirements of each patient. Advanced materials, such as carbon fiber-reinforced polymers and biocompatible resins, are utilized to enhance the strength and comfort of prosthetics. Additionally, 3D printing allows for the integration of aesthetic elements, enabling the creation of prosthetics that closely resemble natural limbs, thereby improving user acceptance and psychological wellbeing (Castro-Franco et al., 2024; Manero et al., 2019).

### 4.4 Tissue engineering scaffolds

In the field of regenerative medicine, 3D-printed tissue engineering scaffolds are essential for bone regeneration and repair. These scaffolds, fabricated from biocompatible and biodegradable materials, provide a temporary structure that supports the growth and differentiation of bone cells (Chung et al., 2020; Su et al., 2021). Materials such as hydroxyapatite and bioactive ceramics are commonly used due to their osteoconductive properties, which facilitate bone in-growth and integration with existing bone tissue. The ability to precisely control the porosity and architecture of 3D-printed scaffolds allows for the mimicry of natural bone structures, promoting vascularization and enhancing the overall effectiveness of the scaffold (de Carvalho et al., 2024; Ielo et al., 2022). To facilitate the design of scaffolds with optimal porosity and architecture, researchers have employed various computational and experimental approaches. For instance, finite element analysis (FEA) can model mechanical stresses within scaffolds that feature different pore sizes and interconnectivities, allowing for a balance between structural stability and nutrient diffusion (Seraji et al., 2024; Li et al., 2022). Topology optimization algorithms further refine the internal geometry according to specific design objectives, such as maximizing bone ingrowth or enhancing vascularization. Additionally, computational fluid dynamics (CFD) simulations help predict fluid flow patterns within the scaffold, ensuring adequate transport of oxygen and other nutrients essential for cell viability (Foroughi et al., 2024). While these methods are rooted in advanced engineering principles, they provide valuable insights that clinicians can utilize in selecting or customizing scaffold designs, ultimately aiming to replicate native bone characteristics and improve implantation outcomes.

Currently, FDA-approved cell-free 3D printing technology is available for large-scale production. The emerging 3D bioprinting methods can solve the above problems. FDA-approved cell-free 3D printing technologies (e.g., Ossiform™ and Cerabone) are already available for large-scale production. In contrast, emerging 3D bioprinting methods integrate living cells and/or bioactive components into printed constructs, potentially overcoming the limitations of purely structural scaffolds. However, these advanced approaches remain in relatively early stages, with most studies focused on proof-of-concept experiments *in vitro* or in small animal models (Zhao et al., 2024). Promising future applications include anatomically customized implants for non-load-bearing bones, intermediate templates for large-scale bone regeneration, and minimally invasive *in vivo* bioprinting for immediate defect repair. Despite ongoing research, the number of active clinical trials is extremely limited. Osteoplug™, a bioresorbable PCL burrhole cover introduced in 2006 by the National University Hospital in Singapore and later approved by the US FDA, has demonstrated favorable vascularization and osseous integration, ultimately leaving no permanent foreign material (Toh et al., 2022). Encouragingly, Hao et al. recently reported the first clinical use of a 3D bioprinted “active bone” scaffold—combining a polycaprolactone/β-tricalcium phosphate (PCL/β-TCP) composite with autologous platelet-rich plasma (PRP)—to repair a left tibial defect (Hao et al., 2023). Recent advancements in bioprinting have further enabled the incorporation of living cells and growth factors directly into the scaffolds, significantly enhancing their regenerative capabilities.

### 4.5 Recent clinical advancements in 3D printing for orthopedic surgery

Recent years have witnessed promising results from patient-specific 3D-printed implants tailored to address severe bone deficiencies, deformity corrections, and arthrodesis procedures. In one series of 15 consecutive patients, each receiving a customized 3D-printed implant cage, clinicians observed significant improvements in pain scores and functional outcomes, ultimately achieving an overall clinical success rate of 87%. Similarly, custom 3D-printed titanium truss arthrodesis implants have emerged as a viable salvage for failed total ankle replacements, providing enhanced stability and patient satisfaction. Beyond implant design, 3D printing has revolutionized the fabrication of ankle–foot orthoses (AFOs). Conventional labor-intensive casting methods often yield suboptimal fit and comfort; by contrast, 3D-printed AFOs seamlessly integrate each individual’s biomechanical requirements and have shown favorable outcomes in patients with plantar fasciitis (Dekker et al., 2018; Lewis et al., 2024; Xu et al., 2019). More recently, 3D-printed acetabular cups have also been reported to improve hip stability and alleviate pain in a small cohort of patients with hip joint defects (Wan et al., 2019). Oldhoff et al. addressed the choice between pre-contoured conventional implants and patient-specific 3D-printed implants for distal radius corrective osteotomies. They found both methods to yield accurate corrections, suggesting that implant selection should be guided by resource availability and preoperative implant fitting rather than accuracy alone (Oldhoff et al., 2024). In another study, Sun reported successful use of 3D-printed artificial vertebrae for multi-segment total *en-bloc* spondylectomy, with radiographs confirming precise fit and stable fixation (Sun et al., 2022). Meanwhile, Thayaparan employed an SLS-printed titanium posterior fixation implant to treat unilateral C1-C2 arthropathy in three patients, observing no screw misplacement or neurovascular complications. They further described a patient-specific titanium implant printed via SLM for occipitocervical fixation; at 6 months, patients showed symptom resolution, satisfactory alignment, and no evidence of loosening, settling, fracture, or implant migration (Thayaparan et al., 2020). In addition, disposable, patient-specific surgical guides produced via 3D printing offer simplified sterilization requirements and reduce additional instrument-related costs without compromising procedural efficiency (Attard et al., 2019). Collectively, these clinical examples underscore the growing utility of additive manufacturing in delivering patient-specific solutions that address complex orthopedic challenges.

**FIGURE 2:**
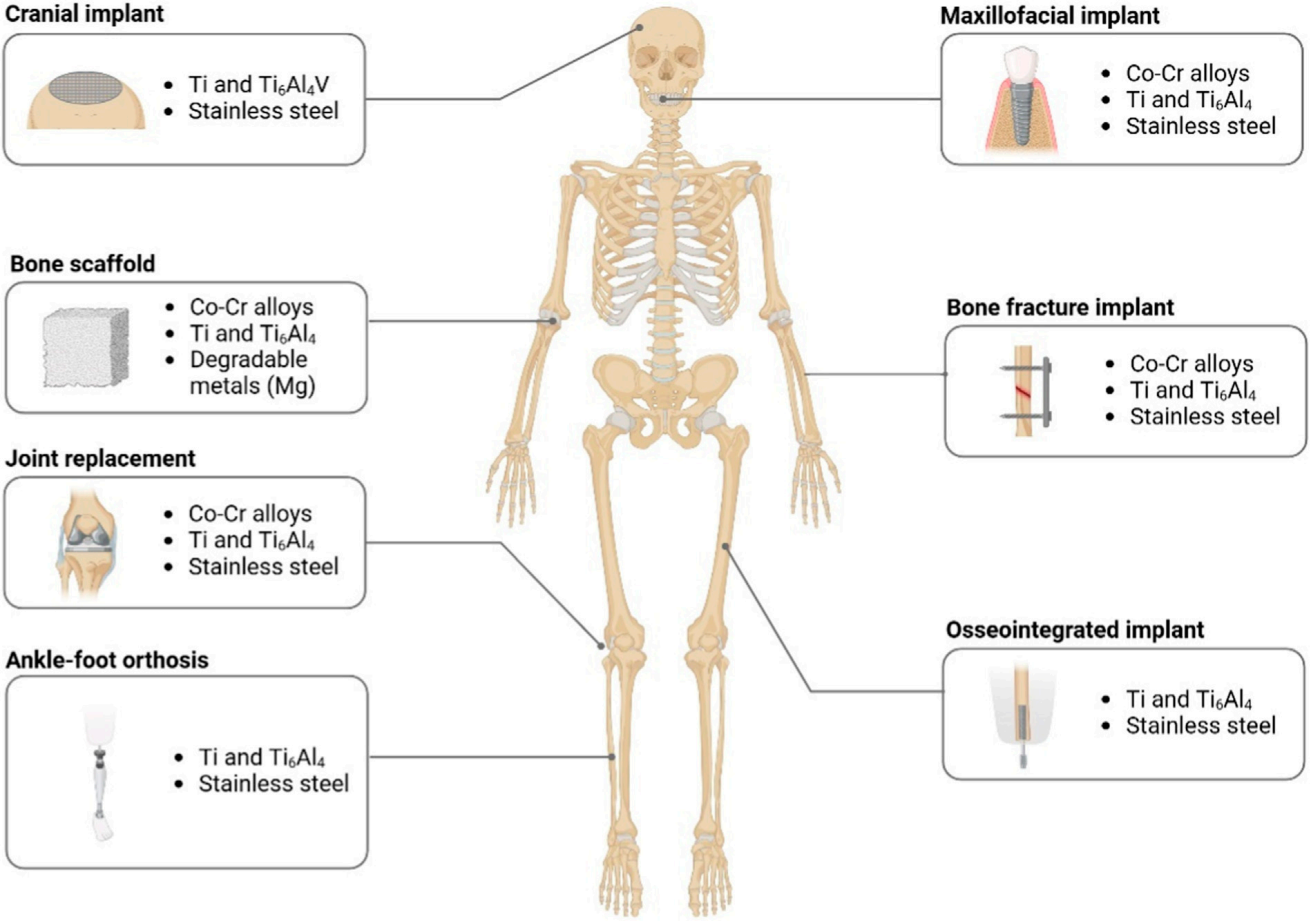
Overview of advanced 3D-Printed orthopedic implants and devices.

Schematic representation of various regions in which 3D printing has been successfully employed to create custom orthopedic implants, prosthetics, and braces. Examples include cranial implants (e.g., titanium meshes), maxillofacial implants (dental or mandibular), bone scaffolds for large defects, bone fracture implants, joint replacement prosthetics, ankle–foot orthoses (AFOs), and osseointegrated limb prostheses. Materials such as cobalt–chromium (Co–Cr) alloys, titanium alloys, stainless steel, and degradable metals (e.g., magnesium) are frequently used, depending on the site-specific mechanical and biological requirements (Figure 2).

## 5 Challenges and future perspectives

### 5.1 Technical and clinical challenges

Despite the significant advancements in 3D printing technologies and materials for orthopedic applications, several technical and material challenges persist. One of the primary technical hurdles is achieving the desired mechanical properties and structural integrity in 3D-printed implants. Although a few short-term case series suggest that 3D-printed implants can achieve promising early osseointegration and mechanical stability, there is a notable scarcity of large-scale, long-term clinical studies specifically tracking failure modes and implant survival over multiple years. This gap in the literature highlights the need for expanded prospective trials that can validate initial findings and elucidate factors influencing implant longevity—such as design optimization, manufacturing consistency, and patient-specific loading conditions. Given the current limitations, more extensive clinical evidence is required to solidify the role of 3D-printed orthopedic implants in routine practice, especially for high-demand, load-bearing applications (Deng et al., 2023). While materials like titanium alloys and PEEK offer excellent strength and biocompatibility, replicating the complex hierarchical structures of natural bone remains difficult (Abdel-Hady Gepreel and Niinomi, 2013). Additionally, the precision and consistency of 3D-printed parts can be affected by factors such as layer adhesion, residual stresses, and anisotropy, which may compromise the reliability and longevity of implants. In recent years, imaging and modeling technologies have evolved significantly, facilitating the precise tailoring of implants in orthopedic procedures. High-resolution imaging modalities, such as multi-slice CT and MRI, enable surgeons and engineers to obtain detailed anatomical data and accurately identify bone defects. Advanced segmentation software, often powered by AI-driven algorithms, refines these imaging datasets into highly accurate 3D reconstructions, expediting the design of patient-specific implants. Virtual surgical planning (VSP) then integrates these models with computer-aided design (CAD) tools and finite element analysis (FEA) packages, allowing developers to simulate mechanical stresses, optimize implant geometry, and predict long-term performance before manufacturing. By streamlining the workflow from imaging to implant fabrication, these technologies not only minimize manual errors and intraoperative guesswork but also ensure that final implants closely match each patient’s unique anatomy and functional needs (Pinto-Coelho, 2023). Building on these imaging and modeling advancements, AI-driven design and computational modeling hold considerable promise for further accelerating innovation. Machine learning techniques can rapidly evaluate multiple implant geometries, materials, and infill patterns against performance criteria such as mechanical stability, biocompatibility, and post-surgical recovery times. Generative design algorithms can then propose novel architectures, sometimes exceeding human intuition, by balancing factors like porosity, load distribution, and weight. Furthermore, real-time feedback loops—integrating patient-specific data from sensors or intraoperative imaging—could dynamically refine implant designs even during surgery. As these methods mature, they may significantly reduce development costs, enable more efficient clinical workflows, and ultimately deliver custom-tailored orthopedic solutions that better withstand physiological demands, especially in complex or high-load scenarios.

An important challenge in 3D-printed implants is anisotropy, which arises due to the layer-by-layer fabrication process, often resulting in weaker interlayer bonding compared to in-plane properties. Optimizing build orientation is one straightforward approach to mitigating this issue; by aligning critical load-bearing axes with the layers, researchers can reduce the stress at interlayer interfaces and improve structural integrity. Additionally, multi-axis or robotic printing is gaining traction, allowing complex deposition paths from multiple angles to create more continuous fiber or material flow. This multi-directional strategy enhances mechanical isotropy by distributing stress more uniformly throughout the printed construct (Allum et al., 2020).

Another promising advancement involves fiber-reinforced composites, where either chopped or continuous fibers (e.g., carbon, glass) are embedded into a polymer matrix. These fibers significantly strengthen the interlayer region, reducing shear failures under fatigue loading. Post-processing treatments such as hot isostatic pressing (HIP) or thermal annealing help densify the printed part, improving cohesion between layers and minimizing voids. Likewise, chemical infiltration with resins or metals can reinforce porous regions and increase overall mechanical robustness.

Recent computational methods further support strategies to counter anisotropy. Finite element analysis (FEA) and topology optimization help researchers identify weak points and evaluate various build orientations before manufacturing, while AI-driven design can quickly iterate through multiple parametric scenarios to optimize infill patterns, layer thicknesses, and printing orientations. Ultimately, the combination of advanced hardware, careful post-processing, and computational modeling holds considerable promise for producing orthopedic implants that maintain mechanical reliability under complex, dynamic loads—a requirement that is particularly critical for load-bearing applications such as spine, hip, and knee reconstructions.

Material limitations also pose challenges; for instance, ceramics, though highly biocompatible and wear-resistant, are inherently brittle and prone to fracture under high-stress conditions, restricting their use in load-bearing applications without adequate reinforcement. Furthermore, the integration of multiple materials within a single implant to mimic the composite nature of bone tissue adds another layer of complexity, requiring sophisticated multi-material printing techniques that are still under development. The production of personalized 3D-printed implants and scaffolds involves sophisticated hardware, specialized materials (e.g., bioinks, titanium powders), and dedicated technical expertise. Such requirements often translate into higher costs and can limit accessibility in low-resource healthcare settings. Ensuring scalability while maintaining product quality is a persistent challenge. Importantly, the dynamic mechanical performance of 3D-printed composites is critical for load-bearing orthopedic applications. While many printed scaffolds and implants can approximate or even exceed the compressive strength and elasticity of native bone, fewer studies have explored their fatigue resistance and long-term durability under physiologically relevant cyclic loading (Bakhtiari et al., 2023). Factors such as porosity, material composition, and interfacial bonding can significantly influence how these composites behave over repeated stress cycles, impacting implant longevity and patient outcomes. Future research focusing on comparative fatigue tests, failure modes, and *in vivo* cyclic loading conditions will be essential to ensure that 3D-printed composites meet the rigorous mechanical demands of clinical practice.

### 5.2 Regulatory complexities and standardization

Regulatory and standardization issues present additional barriers to the widespread adoption of 3D-printed orthopedic implants (Witowski et al., 2018). The customization capabilities of 3D printing necessitate individualized approval processes, as each implant may vary in design and material composition. This variability complicates the establishment of universal standards and regulatory frameworks, making it challenging to ensure consistent quality and safety across different manufacturers and applications. Moreover, the lack of standardized testing protocols for 3D-printed materials and implants impedes the validation of their long-term performance and biocompatibility (Morrison et al., 2015; Ricles et al., 2018). Regulatory bodies are still evolving their guidelines to keep pace with rapid technological advancements, and the absence of clear regulations can delay the clinical translation of innovative 3D-printed orthopedic solutions. Addressing these regulatory challenges requires collaborative efforts between industry stakeholders, regulatory agencies, and academic institutions to develop comprehensive standards that encompass the unique aspects of additive manufacturing in orthopedics.

From a regional perspective, the United States Food and Drug Administration (FDA) has issued guidance documents on “Technical Considerations for Additive Manufactured Medical Devices,” which emphasize risk-based assessments, process validation, and mechanical testing tailored to 3D-printed products. These guidelines acknowledge that fully customized implants may not fit into traditional review pathways, prompting manufacturers to seek either *de novo* classification or investigational device exemptions (IDEs) if devices carry significant patient risk. Similarly, in the European Union, 3D-printed orthopedic implants often undergo assessment by Notified Bodies under the European Medical Device Regulation (MDR), focusing on quality management, post-market surveillance, and clinical evidence. However, the MDR does not yet provide explicit standards for additive manufacturing, leaving manufacturers reliant on general device regulations and *ad hoc* evaluations. This can introduce variability in timelines and requirements, particularly for patient-specific or cell-laden devices (Pettersson et al., 2024). In Japan, the Pharmaceuticals and Medical Devices Agency (PMDA) generally follows a classification system based on risk level, but the agency is increasingly aware that traditional frameworks may not adequately capture the complexity of custom 3D-printed implants. Meanwhile, the National Medical Products Administration (NMPA) in China has recently introduced guidelines to manage personalized orthopedic implants, requiring rigorous device testing protocols and demonstration of reproducible manufacturing processes. Despite these efforts, differences in regulatory detail, clinical evidence requirements, and inspection procedures persist across regions (Jin et al., 2022).

Addressing these regulatory challenges calls for collaborative efforts between industry stakeholders, regulatory agencies, and academic institutions to develop comprehensive standards that encompass the unique aspects of additive manufacturing in orthopedics. Encouragingly, consortia such as the Additive Manufacturing for Medical Research (AM-MR) initiative in the U.S. and similar joint endeavors in the EU and Asia are beginning to tackle these topics. Continued cross-border dialogue can help harmonize regulatory expectations and create globally accepted benchmarks for safety and efficacy. By aligning such standards, it may become easier and more efficient to bring new 3D-printed orthopedic devices to international markets without compromising patient safety or product quality.

Achieving consistent outcomes across multiple print batches is crucial for clinical acceptance. Variations in printer calibration, powder particle size, printing parameters (temperature, pressure, speed), and post-processing protocols can all influence the final mechanical and biological properties of 3D-printed products. Ensuring uniformity in scaffold structure and composition is particularly challenging for multi-material or cell-laden printing strategies. While short-term benefits have been demonstrated in preliminary studies, longer-term data on implant stability, potential inflammatory responses, and integration with host tissue are still limited. Adequate preclinical and clinical evaluations are necessary to confirm the safety of both novel materials (e.g., shape memory polymers, superelastomers) and novel manufacturing processes.

### 5.3 Future outlook: smart materials and functionally graded designs

Looking ahead, the future of 3D printing in orthopedics is poised to be shaped by innovations in smart materials and functionally graded materials (FGMs). Smart materials, which can respond to environmental stimuli such as temperature, pH, or mechanical stress, hold the potential to create implants that adapt to the dynamic conditions of the human body, enhancing their functionality and integration. For example, bioactive smart materials could release therapeutic agents in response to inflammation, promoting healing and reducing the risk of infection (Ahn et al., 2024). Additionally, FGMs, which possess spatial variations in composition and structure, can more closely mimic the natural gradation of bone tissue, providing tailored mechanical properties that enhance load distribution and reduce stress concentrations. Meanwhile, adopting FGMs also involves important trade-offs in cost and manufacturing complexity compared to uniphasic implants. Producing graded architectures often requires specialized multi-material printing systems or sequential material deposition processes, which can extend manufacturing times and necessitate more rigorous quality control protocols. The broader range of materials, combined with variable composition within a single construct, can significantly increase the difficulty and expense of ensuring uniform bonding and consistent mechanical properties. As a result, while FGMs promise superior functionality and patient-specific tailoring, clinicians and manufacturers must weigh the benefits of optimized load distribution and improved osseointegration against higher production costs, extended development timelines, and the need for specialized expertise (Alkunte et al., 2024). These advancements, coupled with improvements in bioprinting technologies that allow for the incorporation of living cells and growth factors, could revolutionize tissue engineering and regenerative medicine in orthopedics (Mahmood et al., 2020; Yan et al., 2020). However, realizing these futuristic applications will require overcoming current technical limitations, ensuring regulatory compliance, and fostering interdisciplinary collaboration to drive innovation forward. While 3D printing holds transformative potential for orthopedic care, its implementation in low-resource settings faces notable challenges. High equipment costs, specialized materials, and the need for technical expertise can limit widespread adoption. One practical approach involves open-source 3D printer designs that rely on locally sourced filaments or bio-based polymers, thereby lowering equipment and material expenses. Shared infrastructure—such as regional fabrication centers—can further distribute overhead and allow multiple clinics or hospitals to benefit from a single advanced printer. Moreover, cloud-based design platforms and AI-guided workflows reduce on-site software and hardware demands, enabling remote experts to assist in implant design and optimization. As these strategies evolve, they may expand the reach of additive manufacturing technologies, delivering personalized orthopedic solutions to populations previously unable to access them. As research progresses, the integration of intelligent and gradient materials with advanced 3D printing techniques promises to deliver more effective, personalized, and resilient orthopedic solutions, ultimately improving patient outcomes and quality of life.

## 6 Conclusion

The integration of 3D printing technologies and diverse materials in orthopedics has significantly advanced the creation of customized implants, surgical guides, prosthetics, and tissue engineering scaffolds, enhancing patient-specific treatments and surgical precision. Despite these advancements, challenges such as achieving optimal mechanical properties, ensuring structural integrity, and navigating regulatory standards remain obstacles to widespread adoption. Looking forward, innovations in smart and functionally graded materials, along with advancements in bioprinting and interdisciplinary collaborations, are expected to address these challenges and further expand the capabilities of 3D printing in orthopedic medicine. As research and development continue, 3D printing holds the promise of delivering more personalized, effective, and durable orthopedic solutions, ultimately improving patient outcomes and quality of life.
